# Dietary intervention for tertiary prevention in head and neck squamous cell carcinoma survivors: clinical and translational results of a randomized phase II trial

**DOI:** 10.3389/fonc.2023.1321174

**Published:** 2024-01-04

**Authors:** Stefano Cavalieri, Eleonora Bruno, Mara Serena Serafini, Deborah Lenoci, Silvana Canevari, Laura Lopez-Perez, Liss Hernandez, Luigi Mariani, Rosalba Miceli, Cecilia Gavazzi, Patrizia Pasanisi, Elena Rosso, Francesca Cordero, Paolo Bossi, Wojciech Golusinski, Andreas Dietz, Primož Strojan, Thorsten Fuereder, Loris De Cecco, Lisa Licitra

**Affiliations:** ^1^ Head and Neck Medical Oncology Department, Fondazione IRCCS Istituto Nazionale dei Tumori, Milan, Italy; ^2^ Department of Oncology and Hemato-Oncology, University of Milan, Milan, Italy; ^3^ Nutrition Research and Metabolomics, Department of Experimental Oncology, Fondazione IRCCS Istituto Nazionale dei Tumori, Milan, Italy; ^4^ Integrated Biology of Rare Tumors Unit, Department of Research, Fondazione IRCCS Istituto Nazionale dei Tumori, Milan, Italy; ^5^ Retired, Milan, Italy; ^6^ Universidad Politecnica de Madrid-Life Supporting Technologies Research Group, ETSIT, Madrid, Spain; ^7^ Biostatistics for Clinical Research Unit, Fondazione IRCCS Istituto Nazionale Tumori, Milan, Italy; ^8^ Clinical Nutrition Unit, Fondazione IRCCS Istituto Nazionale dei Tumori, Milan, Italy; ^9^ Department of Computer Science, University of Torino, Torino, Italy; ^10^ Department of Head and Neck Surgery, Poznan University of Medical Sciences, Poznan, Poland; ^11^ Department of Otolaryngology, Head and Neck Surgery, Universitätsklinikum, Leipzig, Germany; ^12^ Department of Radiation Oncology, Institute of Oncology Ljubljana, Ljubljana, Slovenia; ^13^ Department of Medicine I, Medical University of Vienna, Vienna, Austria

**Keywords:** head and neck cancer, cancer survivors, diet habits, tertiary cancer prevention, translational research, questionnaires, food-derived miRNAs

## Abstract

**Background:**

There is a strong need for preventive approaches to reduce the incidence of recurrence, second cancers, and late toxicities in head and neck squamous cell carcinoma (HNSCC) survivors. We conducted a randomized controlled trial (RCT) to assess a dietary intervention as a non-expensive and non-toxic method of tertiary prevention in HNSCC survivors.

**Methods:**

Eligible participants were disease-free patients with HNSCC in follow-up after curative treatments. Subjects were randomized 1:1 to receive a highly monitored dietary intervention plus the Word Cancer Research Fund/American Institute for Cancer Research recommendations for cancer prevention (intervention arm) or standard-of-care recommendations (control arm). The planned sample size for the event-free survival evaluation (primary endpoint) was not reached, and the protocol was amended in order to investigate the clinical (nutritional and quality-of-life questionnaires) and translational study [plasma-circulating food-related microRNAs (miRNAs)] as main endpoints, the results of which are reported herein.

**Results:**

One hundred patients were screened, 94 were randomized, and 89 were eligible for intention-to-treat analysis. Median event-free survival was not reached in both arms. After 18 months, nutritional questionnaires showed a significant increase in Recommended Food Score (p = 0.04) in the intervention arm vs. control arm. The frequency of patients with and without a clinically meaningful deterioration or improvement of the C30 global health status in the two study arms was similar. Food-derived circulating miRNAs were identified in plasma samples at baseline, with a significant difference among countries.

**Conclusion:**

This RCT represented the first proof-of-principle study, indicating the feasibility of a clinical study based on nutritional and lifestyle interventions in HNSCC survivors. Subjects receiving specific counseling increased the consumption of the recommended foods, but no relevant changes in quality of life were recorded between the two study arms. Food-derived plasma miRNA might be considered promising circulating dietary biomarkers.

## 1 Introduction

Survival in patients with loco-regionally advanced [T3–T4 and/or N1–N3 according to the eighth edition of American Joint Committee on Cancer/Union for International Cancer Control (AJCC/UICC) classification] head and neck squamous cell carcinoma (HNSCC) has not substantially improved in the last decades despite advances in tumor detection and management. Moreover, the lifetime risk of a second primary tumor in patients with HNSCC ranges from 10% to 20% (1).

The most recent GLOBOCAN update (2020) reported that 364,339 patients with head and neck cancer (excluding nasopharyngeal and salivary gland cancers) died yearly worldwide (2). Mortality depends on the primary HNSCC site (3) and country where patients are diagnosed and treated. For instance, even within Europe, there are significant disparities in incidence and cancer-related mortality (4).

Epidemiological studies demonstrated the crucial role of cancer prevention in reducing global cancer mortality (5). Lifestyle habits have a substantial impact in this sense. Smoking exposure (6, 7), HPV-persistent infection (8), and alcohol consumption (9) are known to be among the main etiologic factors that contribute to HNSCC development and prognosis. For instance, according to the systematic review of the World Cancer Research Fund [World Cancer Research Fund International Continuous Update Project (WCRF CUP)], there is strong evidence that consuming alcoholic drinks increases the risk of oral cancer (10). On the other hand, many studies worldwide provided proof of the inverse association between the consumption of vegetables and fruits and the risk of developing HNSCC (11). The WCRF CUP report showed limited evidence that consuming non-starchy vegetables is associated with a lower incidence of HNSCC. In particular, interest has increased in healthy dietary habits, with the consideration of specific diets as tools to improve wellness in cancer prevention (10). Taken together, the results of the International Head and Neck Cancer Epidemiology consortium support a beneficial effect of a dietary pattern based on foods rich in antioxidant vitamins and fiber, like fruit and vegetables, and a detrimental impact of a pattern based on animal products and cereals or high–glycemic index foods on laryngeal cancer risk (12). The American Cancer Society Head and Neck Cancer Survivorship Care Guideline recommends that HNSCC survivors should be counseled to “achieve and maintain a healthy weight,” “maintain a healthy weight for those at risk for cachexia” through nutrition strategies, or, if overweight or obese, “to limit consumption of high-calorie foods and beverages and increase physical activity to promote and maintain weight loss” (13). The vital need for specific nutritional recommendations is particularly significant for HNSCC survivors, who often suffer from relevant long-term and persistent treatment-related adverse events, notably xerostomia, teeth damage, neck fibrosis, late dysphagia, aspiration, etc. (14). These sequelae negatively impact on swallowing function, adequate nutrition, and, ultimately, global quality of life (QoL) (15).

In addition to reducing exposure to risk factors (e.g., quitting smoking and limiting alcohol consumption), counseling for adopting a healthy lifestyle should be regarded as the best clinical practice in HNSCC survivors. Nutritional consultations are widely feasible and affordable and may improve survival while reducing late effects in cured patients with HNSCC through a dietary intervention as a non-expensive and non-harmful tool. To explore the impact of a dietary approach in reducing disease recurrences and the occurrence of second tumors in HNSCC survivors, we conducted a phase II randomized controlled trial (RCT) named DietINT.

Emerging evidence has shown that microRNAs (miRNAs) present in food, also named exog-miRNAs, may resist to digestion process and permeate into human bloodstream (16). These food-derived miRNAs may play a role in regulating several biological functions such as gene expression (17, 18) and contribute to characterization of diet style.

Herein, we report the results of the DietINT study and its main exploratory clinical and translational research data.

## 2 Methods

### 2.1 Study design, participants, and ethical approval

This was an open-label, randomized phase II multi-country trial registered on ClinicalTrials.gov portal (NCT02869399).

Inclusion criteria were as follows: age ≥ 18 years; patients with effectively cured stage III and IV (according to the seventh edition of AJCC/UICC) larynx, hypopharynx, oral cavity, or HPV-negative oropharynx squamous cell carcinoma; smoking history > 10 pack-years in HPV-positive oropharyngeal cancer; administration of at least one of the following treatments (from 3 months to 5 years after the completion of curative treatment): surgery, surgery followed by radiation ± chemotherapy, radiation therapy ± chemotherapy, and induction chemotherapy followed by loco-regional treatment; and absence of major dysphagia, with ability to swallow at least a soft pureed diet.

Patients were excluded in the following cases: previous or concurrent cancer distinct in the primary site or histology from the cancer being evaluated in this study; having received non-parotid sparing radiotherapy; patients with severe malnutrition (weight loss ≥ 5% in the last month before enrollment and with a BMI < 20 kg/m^2^); and patients with diabetes in pharmacological treatment.

Eligible patients were centrally randomized (1:1) and assigned to dietary intervention in addition to standard of care (intervention arm) vs. standard of care (control arm). To balance the characteristic distribution between the two trial arms, the randomization process incorporated minimization techniques based on the following factors: center, primary HNSCC site (oral cavity vs. oropharynx vs. larynx/hypopharynx), stage (III vs. IV), sex, smoking history (current, previous > 10 pack-years, and never smoking or <10 pack-years), previous surgery, and grade >2 xerostomy.

The clinical trial was approved by the Ethical Committees of all the participating institutions (the approval of the study sponsor’s Ethical Committee was obtained on 17 April 2015, internal study ID INT 18-15). All study participants signed a written informed consent. The conduction of the clinical study was supported by the TRANSCAN-2 ERA-NET, JTC 2013 call through the National Funding Agencies specified in the Funding information section.

### 2.2 Study intervention

The dietary intervention was scheduled to run for 24 months. The Word Cancer Research Fund/American Institute for Cancer Research (WCRF/AICR) recommendations for cancer prevention (19) represented the basis for the dietary treatment in the intervention arm.

Briefly, participants randomized in the intervention arm were invited:

- to eat mostly food of plant origin, with a variety of relatively unprocessed cereals (grains) and/or pulses (legumes) with every meal, and a variety of non-starchy vegetables and fruits every day;
- to avoid alcoholic drinks, sugary drinks, processed meat, and salt-preserved food;
- to consume energy-dense food and red meat only sparingly;
- to increase consumption of anti-inflammatory foods, such as fish and whole-grain cereals, e.g., brown rice and barley creams;
- to moderate and limit high glycemic and high insulinemic foods, such as refined flour, white bread, and pastries; and
- to increase the polyunsaturated fats component of the diet.

Patients of the intervention arm attended dietary activities with their caregivers once a month during the 2 years of the study. These activities included cooking classes (about specific nutrition topics), community meals, and dietary reinforcement meetings. A nutritionist was always present in the cooking classes to ensure necessary assistance to the cooks. To strengthen their motivation and improve compliance with the dietary intervention, study participants also received counseling at an individual level. At each follow-up visit (on the 6th, 12th, 18th, and 24th month), patients met the nutritionist and discussed adherence to the diet and any issues related to nutritional suggestions.

A basic curriculum, handout materials, and recipes were developed at each study site and adapted to local traditions. Patients of the intervention arm received a monthly newsletter with suggestions about the diet, recipes, the dish of the month, and a reminder about the study’s procedures.

All efforts were spent to build recommendations that could be easy and transferable to each cultural context in the different countries. As HNSCC survivors frequently suffer from xerostomia following radiotherapy, unrefined products were proposed as whole-grain cereal cream, vegetable cream, or pulse hummus, whose preparation was explained during kitchen classes.

An example of the proposed menu is reported in **Supplementary Figure 1**.

Patients in the control arm did not receive specific suggestions concerning diet but standard healthy lifestyle recommendations, which were provided at each follow-up consultation.

### 2.3 Study endpoints

The initial primary clinical objective of the trial was to assess the effectiveness of the dietary intervention in reducing tumor recurrences and second primary tumors [endpoint: event-free survival (EFS)]. However, the planned sample size (120 patients) was not reached. In addition, because of the COVID-19 pandemic, many patients stopped participating in nutritional activities and clinical follow-up. After amending the protocol due to low accrual, we modified the primary endpoint of the study to the following:

Exploratory clinical endpoints:
- adherence to nutritional recommendations through nutritional questionnaires (24-h food frequency diaries) (20), and
- improvement of patients’ QoL through QoL questionnaires [European Organisation for Research and Treatment of Cancer (EORTC) QLQ-C30 and H&N35].
Exploratory translational endpoints:
- blood-circulating food-derived miRNA, and
- biological sample biobanking.

Available data and biological samples collected during the study conduction are detailed in **Table 1**.

**Table 1:** Available questionnaires and biological samples.

	Baseline	6 months	12 months	18 months	30 months
**# Patients**	89	78	63	56	46
Quality-of-life questionnaires					
**QLQ C30**	48	40	27	18	14
**QLQ H&N35**	64	55	32	22	15
Nutritional assessments					
**Nutritional questionnaires**	58	49	43	33	22
**Daily activity**	33	26	22	15	9
Food-circulating miRNA profiling					
**Plasma**	59	46	36	30	19
Biobanking					
**Plasma ctDNA**	59	46	36	30	19
**Saliva microbioma profiling**	60	43	36	30	26
**Saliva RNA**	48	35	20	27	19
**Saliva DNA methylation**	57	41	36	33	20

### 2.4 Exploratory clinical endpoints

#### 2.4.1 Nutritional assessment: 24-h food frequency diary

The 24-h food frequency diary (paper-based) contained a list of 65 food or food items, without any information on the portion size or weight or on the recipes. Participants had to indicate whether, on the previous day, they had eaten or not eaten the specified food at breakfast, lunch, dinner, and breaks. The list of food was organized into the following six food groups:

- drinks, milk, and dairy products;
- sweets and confectionery;
- bread and cereals;
- meat, fish, eggs, and meat substitutes;
- legumes, vegetables, fresh, and dried fruit; and
- sauces, animal, and vegetable fats.

Participants had to complete the 24-h food frequency diary at baseline (before any dietary recommendations) and at each follow-up visit (on the 6th, 12th, 18th, and 24th month).

#### 2.4.2 Quality-of-life questionnaires

EORTC C30 and H&N35 QoL questionnaires were collected at the same time points described for the nutritional assessment (at baseline and at each follow-up visit). Further details about the collection (paper-based) of QoL questionnaires EORTC QLQ-C30 and H&N35 are described in **Supplementary Methods**.

### 2.5 Exploratory translational endpoints

Our translational aim was to describe at baseline the food-derived miRNAs in the patient population included in this phase II study as potential biomarkers of different dietetic styles in different countries.

#### 2.5.1 Collection and isolation of circulating miRNA

Blood samples were collected before the nutritional intervention and during the study conduction at each follow-up visit. Blood was stored in potassium ethylenediaminetetraacetic acid, acting as an anticoagulant. Samples were processed within 1 h after collection by centrifugation at 1,500 x g at 4°C for 10 min in each of the center; plasma was then aliquoted in nuclease-free tubes and directly stored at −80°C and shipped to the Fondazione IRCCS Istituto Nazionale dei Tumori (INT, Milan, Italy), where extraction methods and sequencing techniques were centralized. Hemolysis was evaluated according to Appierto et al. (21). Total RNAs were isolated using the miRNeasy Serum/Plasma Advanced Kit (Qiagen) automated on QIAcube, following the manufacturer’s instructions.

#### 2.5.2 Whole-miRNA sequencing

Briefly, for next-generation sequencing of mature miRNAs, we utilized with the Qiaseq miRNA library Kit (Qiagen) by a 3′ ligation, adding a pre-adenylated DNA adapter to 3′ ends of all miRNAs, followed by the 5′ ligation. Then, cDNA was obtained through reverse transcription, allowing additionally the integration of UMIs, and it was purified by cleanup using a streamlined magnetic bead–based method. Libraries were amplified, and unique indexes were added, cleaned up, and controlled for quality and quantity using Tape-station 4200 (Agilent Technologies) and Qubit 4 Fluorometer (Thermo Fisher Scientific). After concentration, calculation libraries were individually diluted at 4 nM; 96 libraries were then pooled together for proceeding with the sequencing on NextSeq 550 Sequencer (Illumina).

#### 2.5.3 Analysis of circulating miRNA data and food-derived species identification

The expected structure of the 75-bp reads comprised 15 bp to 55 bp of small RNA sequence, 19 bp of common sequence, 12 bp of Unique Molecular Identifiers (UMI), and a 21-bp adapter (or part of it to reach 75-bp length). bbduk from bbmap [version 38.96 (22)] and extract function in umi-tools [v. 1.1.2 (23)] were used to trim the reads according to the structure described above. The reads shorter than 16 bp or associated with low-quality values were discarded using fastp [v. 1.1.2 (24)].

Survived reads were mapped on miRNome using the Burrows-Wheeler Aligner (BWA) [v0.7.127 (25)] with default settings. Then, the alignment phase was extended to small non-coding RNA (sncRNA) from Database of Small Human noncoding RNAs (DASHR) database v2.0 and RNA central VII and finally to the whole human genome (version hg38). The unmapped reads were aligned versus exogenous miRNAs collected in miRBase 22. After alignment, the miRNA counts of the same exogenous species were aggregated together. All the analyses were performed using R software version 4.1.3.

### 2.6 Statistical analysis

#### 2.6.1 Survival analysis

Median follow-up was estimated with the reverse Kaplan–Meier method. EFS was calculated as the interval between the date of randomization and the date of last follow-up (for alive and event-free participants) or tumor recurrence, second primary tumors, or death, whichever occurred first. EFS was estimated in the intention-to-treat (ITT) population with the non-parametric Kaplan–Meier method, and survival curves were compared with the log-rank test.

#### 2.6.2 Nutritional assessment

Using the data from 24-h food frequency diaries, we generated groups of *recommended* or *not recommended* foods on the basis of the proposed DietINT recommendations by summing the single food items.

The *recommended foods* included 37 items belonging to the following categories: fruits and vegetables (all kinds of fruit and vegetables except potatoes); whole-grain products (whole bread, whole rice, other whole-grain cereals, unsweetened muesli, and oat flakes); legumes and soy products (legumes and tofu/tempeh); fish and mollusks (fish, mollusks, and crustaceans); nuts and seeds (hazelnuts, almonds, walnuts, nut creams, and oilseeds), and vegetable oils (extra virgin olive oil and seeds oil).

The *not recommended foods* included 23 items belonging to the following categories: sugary beverages (sugary beverages and animal milk), alcoholic drinks (wine, beer, and spirits); sweets and cakes (white sugar, artificial sweeteners, chocolate, candies, biscuits, ice creams, and brioches); refined cereals (white bread, white rice, egg noodles, corn flakes, sweetened muesli, potatoes, mashed potatoes, and French fries); red and processed meats (red meat and processed meat); and dairy products (all kinds of fresh or seasoned cheese including pizza and butter).

White meat, eggs, coffee, unsweetened citrus juices, and unsweetened fruit juices were considered neutral foods (not recommended but not discouraged). Stacked bar charts were used to depict the pattern of the recommended and not recommended food groups at baseline, at 6 and 18 months of dietary intervention.

We constructed a Recommended Food Score/day by putting together all the *recommended* foods and a Not Recommended Food Score/day by putting together all the *not recommended* foods as indicators of compliance to the DietINT dietary recommendations. The Wilcoxon–Mann–Whitney rank sum test was used to compare Recommended Food Score/day and Not Recommended Food Score/day at baseline, 6 months, and 18 months measurements in the two randomized groups.

We analyzed the magnitude of changes in food frequency consumption using the difference (*delta*, Δ) between the 6 months–baseline measurements and 18 months–baseline measurements for each patient in the two groups.

All statistical tests were two-sided and those with a p-value of <0.05 were taken as significant. Analyses were done using the STATA 12 statistical package (StataCorp LP College Station, TX, USA) and R software version 4.1.3.

#### 2.6.3 QoL questionnaires

Details about the analysis of QoL questionnaires on EORTC C30 and H&N35 questionnaires are described in **Supplementary Methods**. The global health status (GHS) was assessed as defined by the EORTC QLQ-C30. To assess variations of GHS, we considered subjects completing at least two questionnaires during their post-treatment follow-up. A drop of at least 10 points and an increase in at least 10 points between two questionnaires were considered clinically meaningful GHS deterioration or improvement, respectively (26, 27). We assessed the frequency of patients with and without a clinically meaningful deterioration or improvement of the C30 GHS in the two study arms, comparing them with Fisher’s exact test.

#### 2.6.4 Blood-circulating food-derived miRNA

To perform principal component analysis (PCA), the transformed matrix is computed by the zero multiplicative replacement (version 1.4.0-1 zCompositions) followed by the centered log-ratio transformation on . PCA was performed using FactorMineR (version 1.34).

## 3 Results

### 3.1 Patient characteristics

**Figure 1:**
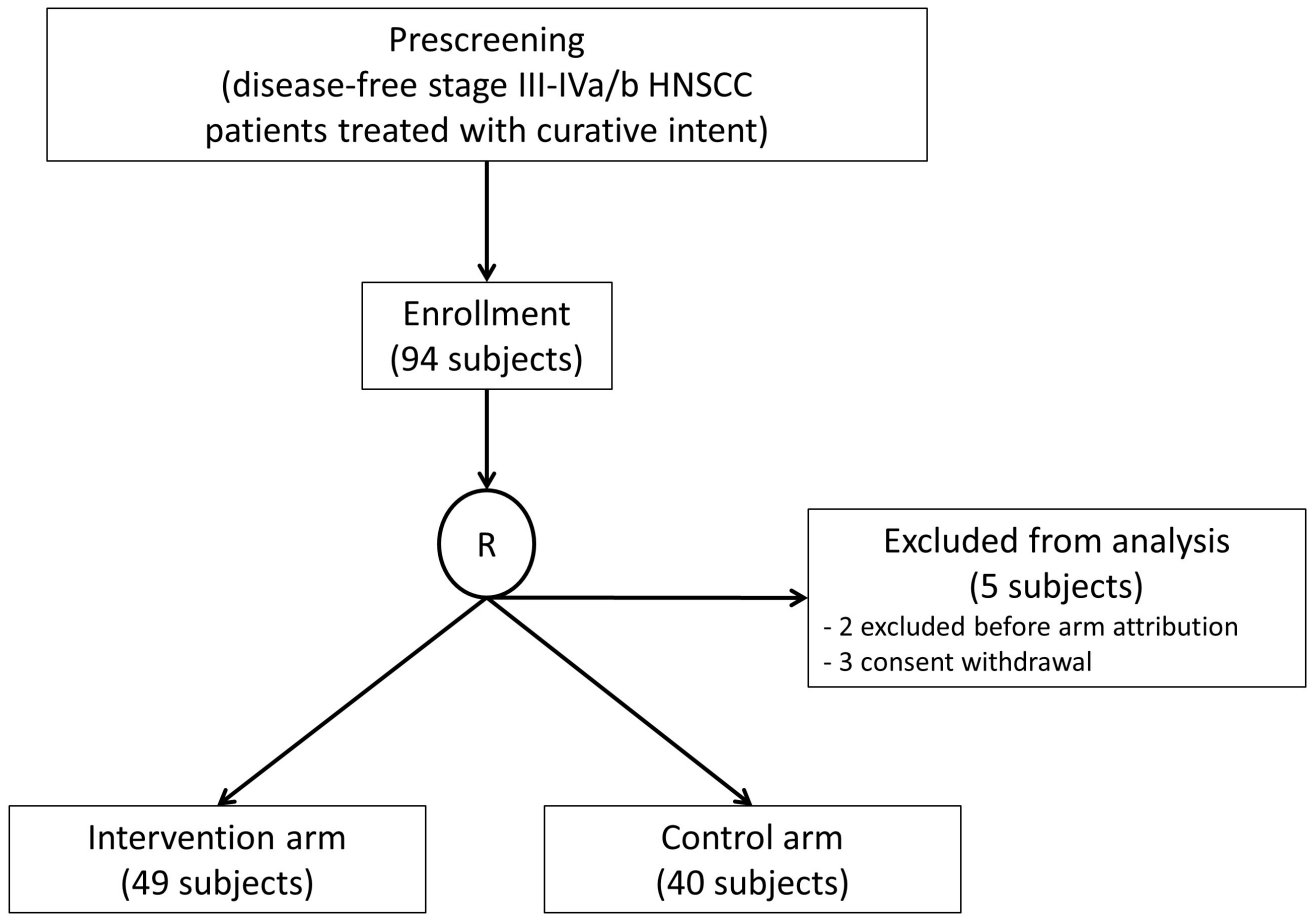
CONSORT diagram.

Accrual started in 2016 in five European cancer centers (Austria, Germany, Italy, Poland, and Slovenia). One hundred patients were screened, 94 were randomized, and 89 were eligible for the ITT analysis: 49 were randomized in the intervention arm, and 40 were randomized in the control arm (**Figure 1**). The accrual distribution between the two study arms for demographic and pre-defined clinical characteristics did not show significant differences (**Table 2**).

**Table 2:** Patient characteristics.

	Total (%)	Intervention arm (%)	Control arm (%)	p-value
No. of patients	89	49 (55%)	40 (45%)	
Sex				
Male Female	71 (80%) 18 (20%)	36 (73%) 13 (27%)	35 (88%) 5 (12%)	0.169*
Age at diagnosis				
Median (range)	60.5 years (43–68)	49 years (43–56)	68 years (65–68)	0.131***
Baseline weight				
Median (range)	70.5 kg (47.6–99)	72 kg (53.3–99)	69 kg (47.6–95)	0.256***
Baseline BMI				
Median (range)	23.81 kg/m^2^ (18.01–35.76)	23.99 kg/m^2^ (18.01–35.76)	23.51 kg/m^2^ (18.6–35.76)	0.204***
Country				
Italy Germany Austria Poland Slovenia	16 (18%) 28 (31%) 1 (1%) 22 (25%) 22 (25%)	8 (16%) 15 (31%) 0 (0%) 12 (24%) 14 (29%)	8 (20%) 13 (33%) 1 (2%) 10 (25%) 8 (20%)	0.785**
Subsite				
Oral cavity Oropharynx HPV-positive HPV-negative Unknown HPV status Larynx/hypopharynx	18 (20%) 49 (55%) 28 (57%) 18 (37%) 3 (6%) 22 (25%)	12 (24%) 24 (49%) 11(46%) 11(46%) 2(8%) 13 (27%)	6 (15%) 25 (63%) 17(68%) 7(28%) 1(4%) 9 (22%)	0.395*
Clinical stage (TNM 7)				
III IVa/b	29 (33%) 60 (67%)	15 (31%) 34 (69%)	14 (35%) 26 (65%)	0.832*
Smoking exposure				
Current Previous ≥10 pack-years Never smoking/<10 pack-years	16 (18%) 60 (67%) 13 (15%)	7 (14%) 32 (65%) 10 (21%)	9 (23%) 28 (70%) 3 (7%)	0.183**
Previous surgery				
Yes No NA	57 (64%) 30 (34%) 2 (2%)	34 (69%) 15 (31%) 0 (0%)	23 (58%) 15 (37%) 2 (5%)	0.211**
Xerostomy				
Grade > 2 Grade ≤ 2	4 (4%) 85 (96%)	2 (4%) 47 (96%)	2 (5%) 38 (95%)	1**

Statistical tests used: *Chi-squared test, **Fisher’s exact test, and ***Mann–Whitney test.

### 3.2 Oncologic outcomes

In the first 3 years of activity, the project suffered slowdowns due to difficulties in participant recruiting; furthermore, because of the COVID-19 pandemic, many patients stopped participating in nutritional activities and clinical follow-ups earlier. Therefore, we limited the follow-up to 18 months as the final observation. Analysis cutoff date was December 2020.

**Figure 2:**
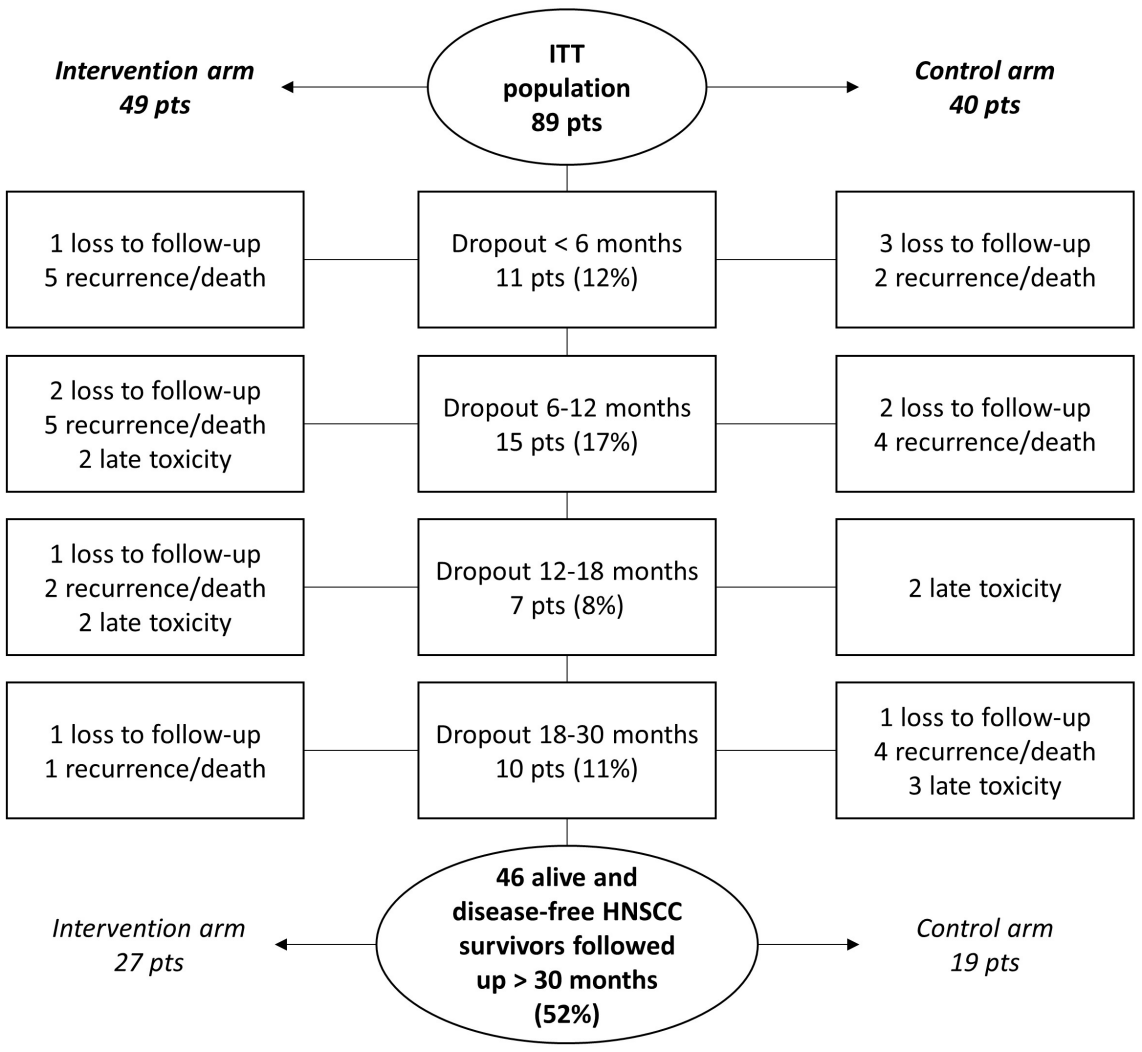
Dropout during the clinical study conduction. pts, patients.

Over a 30-month study duration, 43 of the 89 patients dropped out due to the following reasons: HNSCC recurrence or death (23 of 43; 53.5%), late toxicity (9 of 43; 20.9%), and loss to follow-up (11 of 43; 25.6%) (**Figure 2**).

**Figure 3:**
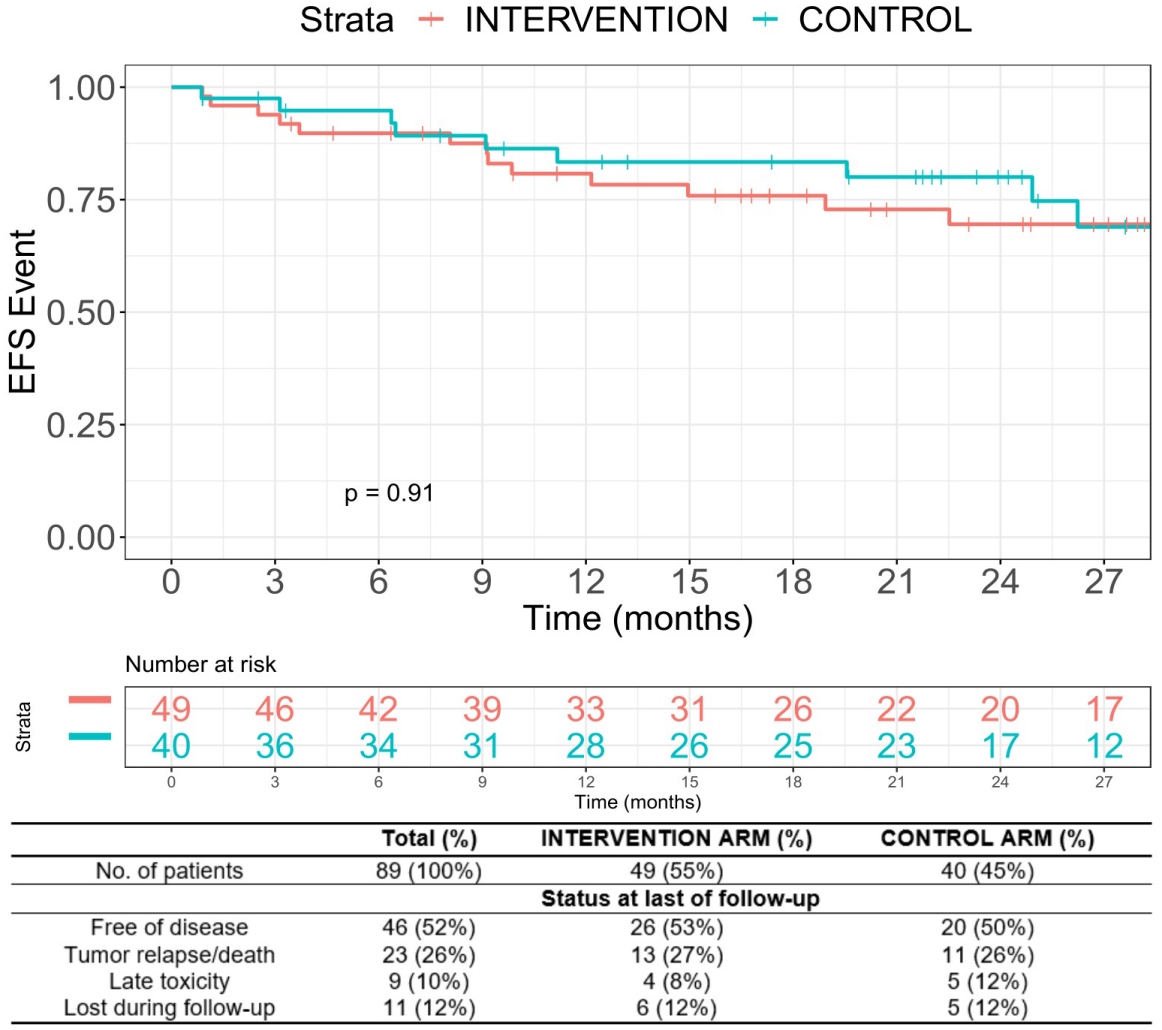
Event-free survival.

Because the accrual goal was not reached and because of the high dropout, the present results are not powered to estimate EFS differences between the two arms. With this caveat, in the ITT population, the median EFS was not reached after a median follow-up of 25.1 months [95% confidence interval (CI), 23.1–29.1). The 3-year EFS was 69.5% in the intervention arm vs. 59.1% in the control arm (p = 0.91, **Figure 3**).

### 3.3 Nutritional assessment

#### 3.3.1 Baseline (at randomization)

The 24-h food frequency diaries were recorded and analyzed at baseline, 6 months, and 18 months.

At baseline (T0), 70 participants (44 in the intervention arm and 26 in the control arm) completed the diary. Less than 20% of participants consumed five or more portions of vegetables and fruits (17.2%) before starting the dietary intervention. As for the *recommended foods*, at baseline, the intervention arm showed slightly higher consumption of vegetables (p = 0.12) and fruits (p = 0.07) and had a slightly higher Recommended Food Score than that in the control group (4.6 ± 3.1 versus 4.42 ± 4.0, respectively; p = 0.70). As for the *not recommended foods*, the control arm showed a slightly higher consumption of refined cereals (p = 0.15), alcoholic drinks (p = 0.06), and processed meat (p = 0.35) with a higher Not Recommended Food Score compared with that in the intervention arm (7.7 ± 3.5 versus 6.2 ± 3.4, respectively; p = 0.07).

#### 3.3.2 Follow-up

The intervention arm showed a significant increase in the consumption of some *recommended foods* such as vegetables and fruits (6 months versus baseline, p = 0.05; 18 months versus baseline, p = 0.09) and nuts and seeds (6 months versus baseline, p = 0.01; 18 months versus baseline, p = 0.08). In general, the intervention arm showed an improvement in the Recommended Food Score at 6 months (6 months versus baseline: 5.3 ± 3.1 versus 4.6 ± 3.1; p = 0.28), with significant results after 18 months (18 months–baseline versus 5.5 ± 3.1 versus 4.6 ± 3.1; p = 0.03).

**Figure 4:**
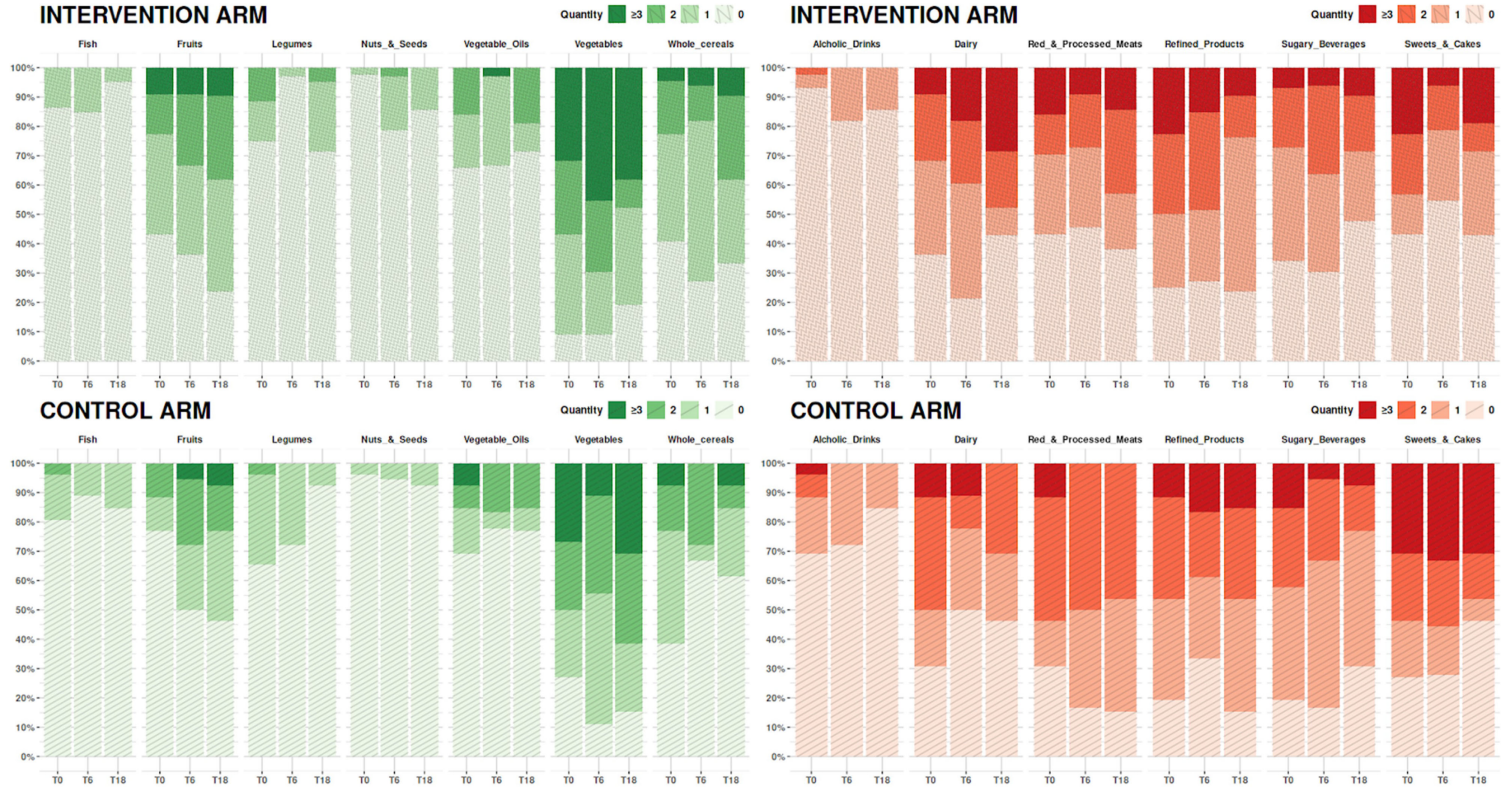
Bar chart representing the food frequencies at baseline (T0), 6 months (T6), and 18 months (T18) according to the food type. On the x-axis, the dietary items considered are reported, and, on the y-axis, the number of subjects according to frequency of the intake is reported.

**Figure 4** shows the analysis of the 24-h food frequency diaries at 6 months (T6) and 18 months (T18) in the intervention and control arms.

Furthermore, the intervention arm showed a slightly not significant reduction in the Not Recommended Food Score at T6 and a significant decrease in the consumption of sweets and cakes at 6 months (6 months–baseline, p = 0.04; 18 months–baseline, p = 0.20).

The control arm substantially did not substantially modify the consumption of fruit and vegetables, worsening the Recommended Food Score at 6 months while moderately improving it at 18 months. Patients in the control arm did not significantly reduce the consumption of *not recommended foods*. Overall, we did not observe significant differences over time in the Not Recommended Food Score in the control arm.

**Figure 5:**
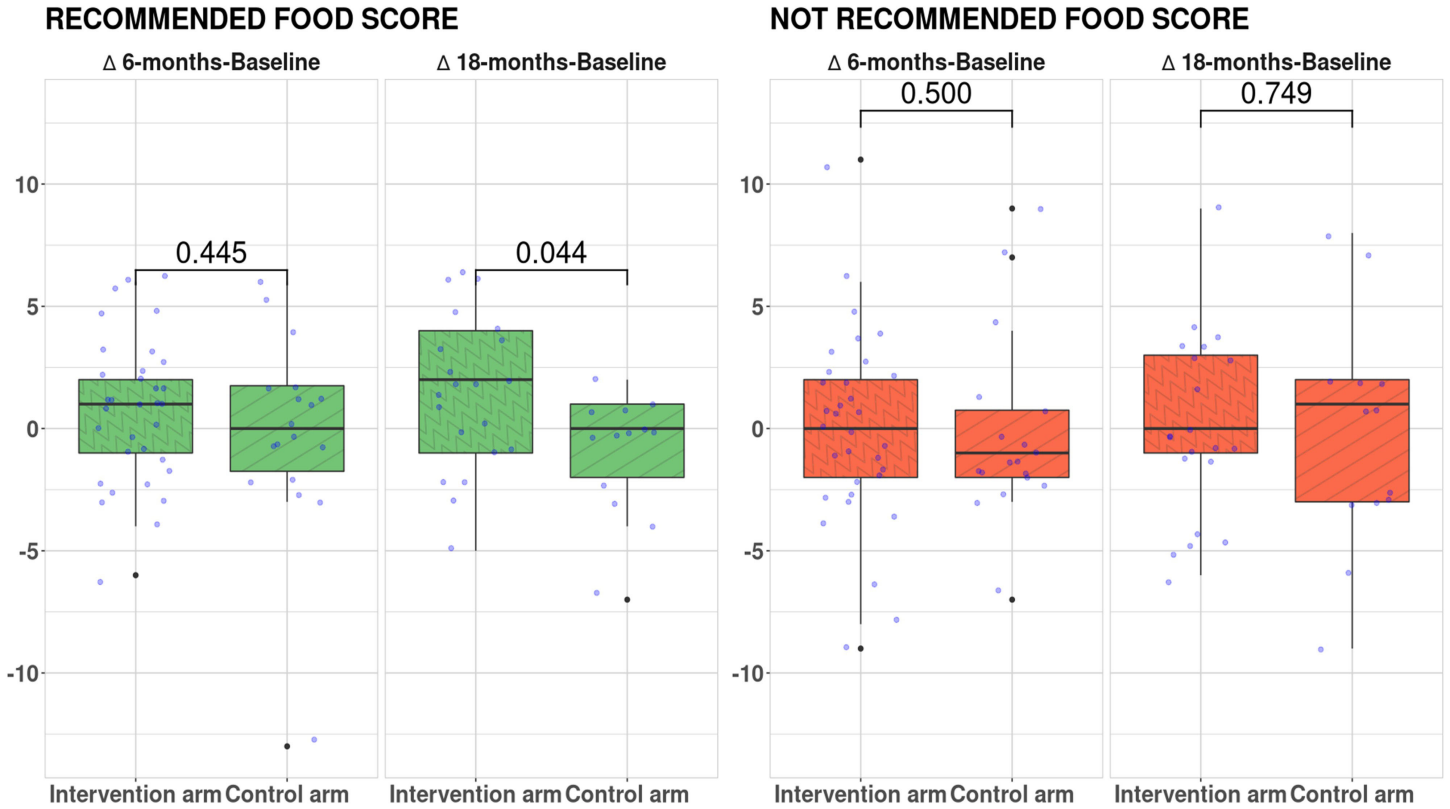
Δ Recommended Food Score and Δ Not Recommended Food Score after 6 months and 18 months of dietary intervention. Statistical tests: two-sided Wilcoxon test.

Boxplots in **Figure 5** show the difference between study arms of the Δ Recommended Food Score and Not Recommended Food Score after 6 months and 18 months of dietary intervention.

Compared with the control, the intervention arm showed a significant increase of Δ Recommended Food Score after 18 months of dietary intervention (p = 0.04). Non-significant differences were reported for Not Recommended Food Score between groups.

QoL data are reported in the **Supplementary Materials**.

### 3.4 Quality of life (EORTC C30 global health status)

QoL questionnaires were completed at baseline in 48 cases for EORTC C30 and in 64 for EORTC H&N35 (**Table 1**). At least two EORTC C30 questionnaires were completed by 38 participants (20 in the intervention arm and 18 in the control arm). A clinically meaningful decrease of patient-reported GHS was observed in 45% (9 of 20) of subjects enrolled in the intervention arm vs. 28% (5 of 18) of those in the control arm (p = 0.327). A clinically meaningful increase of patient-reported GHS was observed in 65% (13 of 20) and 67% (12 of 18) of subjects enrolled in the intervention and control arms, respectively (p = 1.0). Further results on QoL analyses are reported in the **Supplementary Materials**.

### 3.5 Food-derived circulating miRNAs

**Figure 6:**
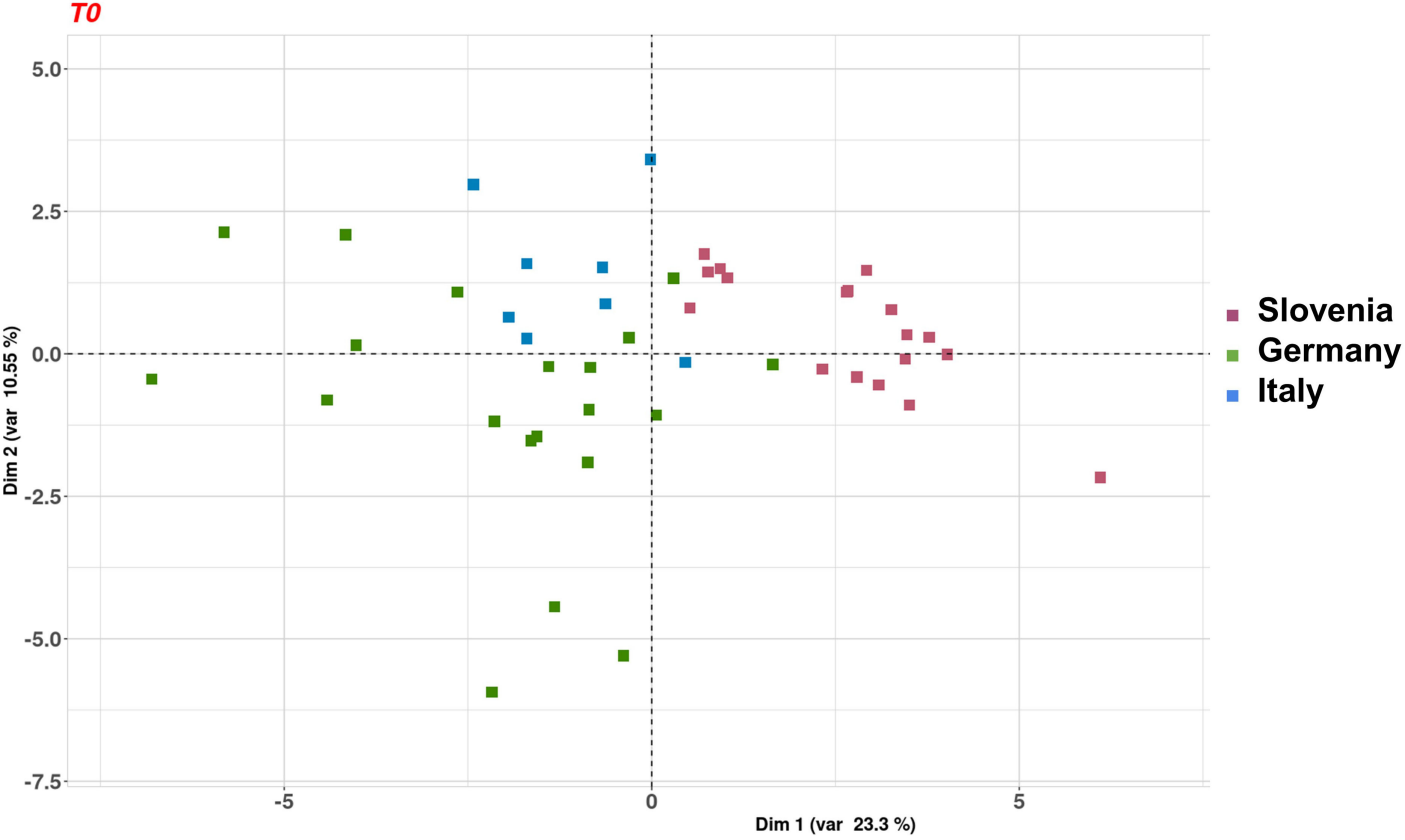
Principal component analysis of the 49 serum samples collected at baseline and with informative miRNA data. Colors represent patients’ country (Germany, green; Italy, blue; and Slovenia, pink).

After quality check by hemolysis, circulating miRNA profiling was performed in plasma specimens at baseline from three countries (i.e., Italy, Germany, and Slovenia). A total of 49 informative samples were included (see details in **Supplementary Results**). Food-derived circulating miRNAs were identified in all plasma samples, but we observed significant differences among patients recruited in different countries. Thus, we decided to evaluated the differences in food consumption in the three European countries at baseline (i.e., at randomization) by PCA. The first two principal components retained 32% of the variance present in the data, and they separated samples by patients’ country of origin (**Figure 6**). Although the percentage of variability is limited, the separation was particularly evident for Slovenian samples compared to the others (Italy and Germany).

Consistently, when we compared the sum of the Recommended Foods from the 24-h food frequency diaries at baseline, we observed a significantly lower consumption of this food group in Germany compared with that in the other countries (Germany = 3.5 ± 2.9 versus other countries = 5.2 ± 3.2; p = 0.03). About not recommended foods at baseline, we observed a worsening score of not recommended foods in Slovenia compared with that in the other countries, even if not significant (Slovenia = 7.4 ± 3.0 versus other country = 6.4 ± 3.7; p = 0.30).

## 4 Discussion

The present reported study is the first prospective randomized dietary trial on patients with HNSCC conducted in five different European countries. Herein, we demonstrated that a European-based dietary intervention is also feasible for HNSCC population, although challenging, and that it can be conducted and assessed over time. However, several difficulties were encountered in starting and reaching a sufficient number of enrolled participants with at least 2 years of follow-up. The study failed its accrual goal, and no conclusions about the effects of diet on oncologic outcomes can be drawn.

In addition to the recruitment obstacles, further barriers have made the DietINT RCT results not suitable to perform efficacy and some translational research analyses: the high dropout in both arms; the difficulty in saliva collection during follow-up, essentially related to the treatment-related xerostomia, reduced the number of available samples for translational research purposes. Another limitation is the unavailability of long-term adverse events, which may have impacted on patients swallowing function, nutritional behaviors, and QoL. On the other hand, a lesson learned from the DietINT experience is that a dietary intervention, although challenging to conduct and thoroughly assessed over time, is feasible. Moreover, this RCT’s exploratory objectives allowed us to obtain relevant observations on the nutrition habits of HNSCC survivors. Indeed, the analyses envisaged for the clinical and translational endpoints were feasible in approximately 60% and half of the enrolled patients, respectively, and the data obtained were informative.

Although our dietary instrument was unable to estimate the patient’s nutrients intake, the 24-h food frequency diaries were sufficient to describe and compare the average consumption of specific foods and dietary patterns. In the DietINT intervention arm, nutritional questionnaires after 6 months showed a compliance to the diet in the intervention vs. the control arm supported by the following: i) a significant improvement in the consumption of fruits and vegetables and whole grains over refined grains; and ii) a slight improvement in consumption of general fish and poultry over red and processed meats, unsaturated over saturated fats, and low-fat over full-fat dairy. These findings suggest that the active dietary intervention and nutritional counseling were effectively working.

We did not find significant differences in terms of improvement or deterioration of patient-reported GHS between the two study arms. We cannot exclude that this observation could be due to the low number of patients completing at least two QoL questionnaires over time (20 in the intervention arm vs. 18 in the control arm).

Notably, apart from the WCRF/AICR recommendations for breast cancer survivors (19), there are no tertiary dietary guidelines for other cancer survivors beyond those recommended for primary cancer prevention. Moreover, literature data are scant about dietary intervention in patients with HNSCC: i) in a meta-analysis of five multi-ethnic and multi-geographic cohort studies, an inverse association between total vegetable consumption before diagnosis and overall mortality was recorded (28); and ii) a strong association was detected for both all-cause and site-specific cancer mortality with post-diagnosis total vegetable intake compared with pre-diagnosis intake (29). The lifestyle modification approach for patients with cancer is still worthy of attention, especially in the patient population with HNSCC, known for being poorly compliant with medications, after having completed highly toxic treatments. In addition, HNSCC survivors frequently present evident chewing and swallowing problems after therapy (14), and nutrition intervention should focus on teaching them how prepare some recommended foods in an easy way that could be relevant.

A drawback in monitoring survivors’ nutrition over time is the lack of objective indicators of adherence to a recommended diet during post-treatment follow-up. Dietary miRNAs represent a new area in food science. Different studies proved the assumption of food-derived exogenous miRNAs depending on various diet schemes, their processing stability, and their detectability in blood specimens (17). In addition, a recent review reported the results from independent groups suggested that these exogenous miRNAs may be functional in organisms (30). Even if the biological samples were homogeneously collected and analyzed at a central laboratory (i.e., at the RCT sponsor site), we observed that food-derived plasma miRNAs significantly differ among patients recruited in the three countries. Therefore, the observed geographical variations could be interpreted as a likely consequence of dietary, lifestyle, and cultural differences among countries, even if belonging to a common European context. This hypothesis is corroborated by the consistent results that were found comparing baseline food frequency diaries at different centers and countries.

On one side, this finding hampered the feasibility of conducting trustable longitudinal observations and comparisons between the two study arms. A time-dependent monitoring between the two study arms should be corrected considering this country-specific heterogeneity. However, because of the significant dropout and the limited number of samples precluded this approach: 21 plasma samples at 6 months (N = 15 in the intervention arm and N = 6 in the control arm) and 17 samples at 18 months (N = 8 in the intervention arm and N = 9 in the control arm).

The function of exogenous miRNAs entering into systemic circulation is still debatable (31). However, different studies proved the assumption of food-derived exogenous miRNAs depending on various diet schemes, their processing stability, and their detectability in blood specimens (17, 32, 33). Therefore, they are now actively being investigated as functional food elements of dietary supplements. The exact implications of the physiological effects of dietary miRNAs entering the bloodstream require further research, both *in vitro* and *in vivo*. On the other hand, we obtained a proof of principle that the obtained information may underlie the role of food-derived plasma miRNAs as valuable, objective, and easily retrievable circulating biomarkers of dietary monitoring in HNSCC survivors and in patients with all-type cancer. The exact implications and importance of circulation food-derived miRNAs as biomarkers require further research. Finally, the trial enabled the collection of a consistent number of different types of samples from each patient. Their programmed analysis could generate a large amount of additional data that will allow us to increase the knowledge of potential HNSCC biomarkers to identify those that a dietary intervention could modulate and to discern better the profile of HNSCC survivors and how to improve their long-term QoL.

## 5 Conclusions

The DietINT RCT represents the first proof-of-principle experience indicating the feasibility of conducting a prospective study based on nutritional and lifestyle interventions in HNSCC survivors. It enabled us to identify critical limitations and possible ways to overcome them to obtain informative results. Nutritional questionnaires showed that subjects receiving specific counseling increased the consumption of recommended foods over time. Through the translational research analysis, the DietINT experience was the first study investigating the miRNome to explore the relationship between circulating exogenous miRNAs and diet intake in HNSCC survivors. Preliminary data at baseline suggested that circulating food-derived exogenous miRNA levels could be promising circulating dietary biomarkers and may be used to identify different dietary regimens; however, further analyses in larger cohorts of patients separated by the Country of origin are needed to confirm this hypothesis.

## Data availability statement

The original contributions presented in the study are included in the article/**Supplementary Material**, further inquiries can be directed to the corresponding author/s.

## Ethics statement

The studies involving humans were approved by Ethics Committee of the research consortium coordinator and the clinical study sponsor, Fondazione IRCCS Istituto Nazionale dei Tumori (local study identifier INT 18-15, approved on the 17th April 2015), Milan, Italy. The study was then approved by the ethical committees of each institution recruiting study participants. The studies were conducted in accordance with the local legislation and institutional requirements. The participants provided their written informed consent to participate in this study.

## Author contributions

StC: Data curation, Formal analysis, Methodology, Validation, Writing – original draft, Writing – review & editing. EB: Conceptualization, Data curation, Formal analysis, Investigation, Methodology, Project administration, Resources, Supervision, Validation, Writing – original draft, Writing – review & editing. MS: Data curation, Formal analysis, Investigation, Methodology, Project administration, Resources, Validation, Writing – original draft, Writing – review & editing. DL: Data curation, Formal analysis, Writing – review & editing. SiC: Data curation, Investigation, Methodology, Project administration, Resources, Supervision, Validation, Writing – review & editing. LL-P: Data curation, Formal analysis, Writing – review & editing. LH: Data curation, Formal analysis, Writing – review & editing. LM: Methodology, Writing – review & editing. RM: Methodology, Writing – review & editing. CG: Supervision, Writing – review & editing. PP: Supervision, Writing – review & editing. ER: Data curation, Formal analysis, Writing – review & editing. FC: Data curation, Formal analysis, Writing – review & editing. PB: Conceptualization, Project administration, Writing – review & editing. WG: Data curation, Investigation, Project administration, Resources, Writing – review & editing, Funding acquisition. AD: Data curation, Investigation, Project administration, Resources, Writing – review & editing, Funding acquisition. PS: Data curation, Investigation, Project administration, Resources, Writing – review & editing, Funding acquisition. TF: Data curation, Investigation, Project administration, Resources, Writing – review & editing, Funding acquisition. LC: Data curation, Formal analysis, Investigation, Methodology, Resources, Writing – original draft, Writing – review & editing. LL: Conceptualization, Funding acquisition, Investigation, Methodology, Project administration, Resources, Supervision, Writing – review & editing.
