# Supplementary material for: Dietary intervention for tertiary prevention in head and neck squamous cell carcinoma survivors: clinical and translational results of a randomized phase II trial
Source: Front Oncol. 2024 Jan 4;13:1321174. doi: 10.3389/fonc.2023.1321174 (PMC10794719; doi:10.3389/fonc.2023.1321174)
Supplement: Supplementary file 1 [file DataSheet_1.docx]

***Supplementary Material***

1. Supplementary Methods
   1. Dietary intervention

Supplementary Figure 1. Example of a DietINT lunch

- 1. Quality of life

Due to the absence of significant differences in outcomes between the intervention and the control arms, quality of life (QoL) data were analyzed pooling together these two populations. The global health status (GHS) was assessed as defined by the EORTC QLQ-C30. To assess variations of GHS, we considered subjects completing at least two questionnaires during their post-treatment follow-up. A drop of at least 10 points and an increase in at least 10 points between two questionnaires were considered clinically meaningful GHS deterioration or improvement, respectively (1,2).

For each scale (EORTC QLQ-C30 and HN35) at each timepoint we calculated the average score and its standard deviation.

The score distribution of the C30 and HN35 scales were assessed in both study arms. To assess for potential imbalances at randomization, we compared the baseline score distribution between the two arms. Since median follow-up (estimated with reverse Kaplan-Meier method) was 25.1 months, we compared the scales at baseline, 6 months and 18 months. For those scales without statistically significant differences at baseline (set as p<0.1), we performed the following comparisons: distribution of the unpaired overall score at 6 months vs baseline in each arm; distribution of the unpaired overall score at 18 months vs baseline in each arm; distribution of the paired overall score between 6 monthsand baseline in each arm; distribution of the paired overall score between 18 months and baseline in each arm. All these comparisons were assessed through the Wilcoxon-Mann-Whitney rank-sum test for continuous variables, Fisher’s exact test for discrete ones. QoL analyses were performed using SAS® OnDemand for Academics.

For differences in scales reaching a statistical significance in at least one arm, we plotted a graph reporting the average values for each arm at the pre-specified timepoints.

- 1. Exploratory translational endpoints

The retained exogenous species associated with at least 10 reads in more than two samples were 32. The resulting count matrix was $M_{spsa}$ with the species in row and samples in column (Supplementary Figure 2).

The 32 species were classified into different categories according to study-based dietary recommendations to analyze the food-derived miRNA. Specifically, 17 species were classified as “Recommended” namely cereals (e.g., soybean, bread wheat, barley and rice), fish (e.g., cod, salmon), fruits (e.g., clementine, orange, muskmelon, strawberry, apple, peach), sunflower as source of acceptable vegetable oils, and vegetables (e.g., asparagus, turnip, artichoke, tomato). Nine species were “Not recommended”, namely five farm animals (e.g., cattle, goat, horse, pig, sheep), cacao and honey as sweets, and potato and maize as refined cereals because of their high carbohydrate content. Six species, namely, peanut, papaya, sorghum, rape, grape, and chicken, were not considered since they were neutral food or their origin was uncertain. The z-score was computed on the aggregated matrix according to the macro categories cereals, fish, fruits, vegetable oil, vegetables, animals, sweets, refined cereal, and unknown.

1. Supplementary results
   1. Exploratory clinical endpoints: Quality of life

EORTC QLQ-C30 questionnaires were available for 50 patients: 38 of them (76%) completed at least two questionnaires during their post-treatment follow-up.

Overall, a significant improvement of GHS during follow-up was observed in 25 patients (66%). The prevalence of patients suffering from a clinically meaningful GHS deterioration was 37% (14 patients). Half of them (7 cases) underwent a subsequent improvement, half had no clinically meaningful recovery during their follow-up.

- - 1. Questionnaires at randomization

Baseline EORTC QLQ C30 were available in 67 cases (36 subjects in the intervention arm, 31 in the control arm), QLQ HN35 in 50 cases (28 in the intervention arm, 22 in the control arm). The EORTC QoL scales being significantly different between arms at baseline were emotional and cognitive functioning for C30. For HN35, a baseline imbalance between arms was observed in the following scales: pain, senses problems, trouble with social eating, social contact problems, teeth problems, sticky saliva, and coughing. All the remaining QLQ C30 and HN35 scales did not differ significantly between arms at baseline (C30: physical functioning, role functioning, social functioning, fatigue, nausea and vomiting, pain, dyspnea, insomnia, appetite loss, constipation, diarrhea, financial difficulties, global health status. HN35: swallowing problems, speech problems, less sexuality, opening mouth, dry mouth, felt ill, pain killers, nutritional supplements, feeding tube, weight loss, weight gain).

- - 1. Questionnaires over time

No significant variations over time were observed in C30 scales. The HN35 scales with significant differences between pre-defined timepoints in the intervention arm and not in controls were opening mouth (Intervention arm: 6 months-baseline, p=0.377; 18 months-baseline, p=0.016. Control arm: 6 months-baseline, p=0.0738; 18 months-baseline, not significant [n.s.]) and nutritional supplements (Intervention arm: 6months-baseline, p=0.0284; 18months-baseline, p=0.0807. Control arm n.s.). The ones differing significantly in the control arm only were dry mouth (Intervention arm: 6 months-baseline, n.s.; 18 months-baseline, p=0.066. Control arm: 6 months-baseline, p=0.0553; 18months-baseline, p=0.0445), and weight gain (Intervention arm: n.s. Control arm: 6months-baseline, p=0.0395; 18 months-baseline, p=0.0627).

The only statistically significant difference between arms of intra-individual variations over time was the presence of feeding tube (6months-baseline in Intervention arm vs Control arm, n.s.; 18months-baseline in Intervention arm vs. Control arm, p=0.033).

- 1. Exploratory translational endpoints

At baseline miRNA were identified in all serum informative samples (49/59 extracted sera). After alignment: the reads shorter than 16 bp or associated with low quality values were discarded (figure 2 = orange); survived reads were mapped on miRNome, Small Human noncoding RNAs database and whole human genome (figure 2 = green). The unmapped reads were aligned versus exogenous miRNAs collected in miRBase 22(Figure 2 = blue).

Supplementary Figure 2: miRNA identified at baseline in all available and informative samples The food derived miRNAs were included and identified in the category other RNA reads (blue color).

1. Supplementary references

1. Osoba D, Rodrigues G, Myles J, Zee B, Pater J. Interpreting the significance of changes in health-related quality-of- life scores. *J Clin Oncol* (1998) **16**:139–144. doi:10.1200/JCO.1998.16.1.139

2. Cocks K, King MT, Velikova G, De Castro G, Martyn St-James M, Fayers PM, Brown JM. Evidence-based guidelines for interpreting change scores for the European Organisation for the Research and Treatment of Cancer Quality of Life Questionnaire Core 30. *Eur J Cancer* (2012) **48**:1713–1721. doi:10.1016/j.ejca.2012.02.059
